# Inadvertent Transarterial Lead Placement in the Left Ventricle and Aortic Cusp: Percutaneous Lead Removal with Carotid Embolic Protection and Stent Graft Placement

**DOI:** 10.1016/s0972-6292(16)30565-4

**Published:** 2012-12-02

**Authors:** Ioanna Kosmidou, Dimitri Karmpaliotis, David E Kandzari, Dan Dan

**Affiliations:** Piedmont Heart Institute, Atlanta, GA, USA

**Keywords:** Pacemaker, defibrillator, lead removal, carotid embolic protection

## Abstract

**Background/Purpose:**

Transarterial lead implantation in the left ventricle or aorta is a rare complication. Percutaneous lead removal is associated with significant thromboembolic and bleeding risk. We present two cases of lead removal from the left ventricle via the left subclavian artery with concurrent carotid embolic protection followed by stent graft placement in the subclavian artery.

**Methods/Results:**

Patient 1 underwent prior pacemaker implant with atrial and ventricular active fixation leads positioned in the right coronary cusp and the left ventricle, respectively. Patient 2 had prior ICD implant with a single active fixation lead positioned in the left ventricular apex. Lead removal was performed in a hybrid operating room. Distal embolic filter wires were deployed in the carotid arteries following anticoagulation. Intravascular ultrasound of the left subclavian artery was performed and as the leads were withdrawn, a covered stent was deployed at the removal site. Final angiography demonstrated no evidence of embolic phenomena. Both patients underwent transvenous lead implantation followed by an uneventful postoperative clinical course.

**Conclusions:**

Transarterial percutaneous lead removal may be safely performed using embolic filter protection of the cerebral circulation and stent graft placement of the arterial entry site.

## Introduction

Inadvertent transarterial lead implantation in the left ventricle or aorta is a rare but potentially serious complication of cardiac rhythm device implantation. Percutaneous lead removal may pose significant thromboembolic and bleeding risk. Rare literature reports describe several different management approaches, including surgical removal or lifelong anticoagulation [1-3]. In cases of transvenous implants with subsequent inadvertent puncture of the ventricular or intratrial septum or placement via a preexisting atrial septal defect, with resultant lead positioning in the LV, transvenous removal has been successfully attempted. However, only rare reports of transarterial lead removal exist. We present two cases of percutaneous lead removal from the left ventricle and/or aorta via the left subclavian artery with a novel method employing carotid embolic protection followed by stent graft placement in the subclavian artery to achieve hemostasis.

## Case 1

A 79 year old male with history of sick sinus syndrome associated with frequent syncopal events underwent dual lead pacemaker implantation with atrial and ventricular active fixation leads. Six months post implant he experienced acute inferolateral ST-segment elevation myocardial infarction complicated by cardiogenic shock and underwent successful primary percutaneous revascularization. During diagnostic coronary angiography the angiographic catheters were identified to engage the previously implanted pacemaker leads. Inadvertent positioning of the atrial and ventricular leads via the left subclavian artery to the right coronary cusp and left ventricular apex, respectively, was confirmed with detailed fluoroscopy and selective subclavian angiography. Pacemaker interrogation confirmed lack of atrial capture and normal ventricular sensing and capture thresholds. Following revascularization, the patient had an uneventful hospital course and was discharged on appropriate medical therapy including antithrombotic therapy with aspirin and thienopyridine. Warfarin was not instituted due to persistent anemia and requirement of dual antiplatelet therapy.

**Figure 1:**
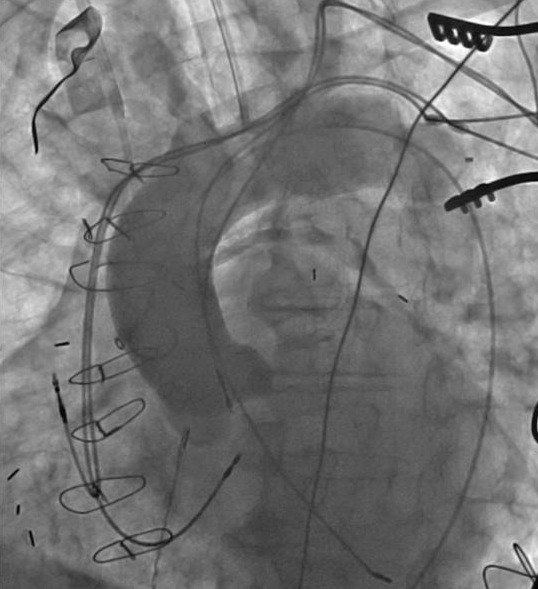
Aortic root aortography demonstrating the initial atrial lead at the right coronary cusp and initial ventricular lead at the LV apical wall. A new pacemaker system is also seen with the atrial lead at the right atrial appendage and ventricular lead at the RV septum.

Given the high risk position of the atrial lead tip in close proximity to the right coronary artery ostium, the patient was electively admitted one month later for transarterial lead removal. Under general anesthesia and in a hybrid operating theater and catheterization laboratory, vascular access was obtained in the right and left femoral arteries and right femoral vein. Prior to systemic anticoagulation, the left subpectoral pocket was opened under sterile conditions, and the subcutaneous tissue was carefully dissected until the leads were visible and freed. Venipuncture was performed using the Seldinger technique, and the left axillary vein was accessed. Two active fixation pacemaker leads were implanted in the right ventricular outflow tract and the right atrial appendage, respectively. Fluoroscopy and aortography were performed demonstrating the dual pacemaker system (Figure 1). Aortic arch angiography demonstrated a type 1 arch and no significant disease in the origin of the great vessels. Using 5-French diameter, 90 cm length guiding sheaths, the right and left common carotid arterieswere selectively engaged over a guidewire. Carotid angiography revealed nonobstructive disease in the intracranial and extracranial portions bilaterally. Unfractionated heparin was administered to achieve an activated clotting time of approximately 350 seconds. A distal embolic filter wire (Spider, EV3 Inc., Plymouth, MN)was then deployed in the distal segment of the both internal carotid arteries. Selective left subclavian angiography was then performed, confirming the pacemaker leads entry point in the subclavian artery, but no critical subclavian arterial stenosis. Unfractionated heparin was administered to achieve an activated clotting time of approximately 350 seconds.

**Figure 2:**
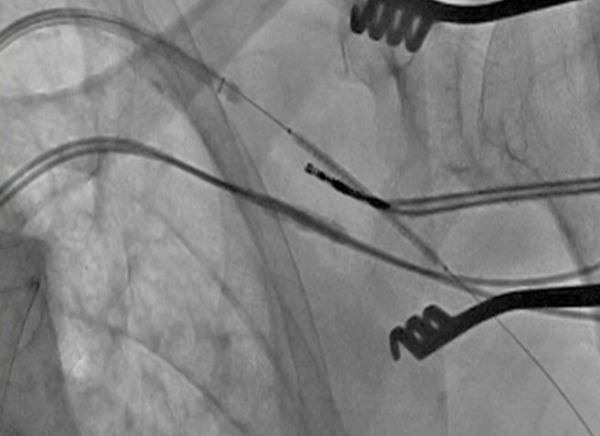
Covered stent deployment at the arterial insertion site during pacemaker lead withdrawal.

Stylets were then advanced through the chronic transarterial leads, and after retracting the active fixation mechanism, gentle traction on the ventricular lead enabled withdrawal without requirement of laser extraction. Similarly, the atrial lead was manually withdrawn from the aortic root, and both leads were carefully pulled back until the tips were in close proximity to the insertion site of the subclavian artery. Over an antegradeguidewire, intravascular ultrasound was performed to ensure appropriate sizing for an 8mm x 60 mm self-expanding nitinol covered stent (Viabahn, W.L. Gore, Flagstaff, Arizona) placement. The stent was delivered and positioned between the proximal and distal insertion site margins and deployed immediately as the leads were withdrawn (Figure 2). Angiography confirmed adequate stent expansion and no contrast extravasation. The distal embolic protection devices were subsequently withdrawn. Following the procedure, the patient remained hemodynamically stable, and recovery was uneventful without neurological or vascular complications.

## Case 2

A 63 year old male with history of ischemic cardiomyopathy and single chamber ICD with a dual coil active fixation lead implanted twelve months prior to presentation, underwent routine echocardiography identifying the lead localized in the aortic root and crossing the aortic valve. The proximal coil was adjacent to the right coronary cusp. The patient had recurrent gastrointestinal bleeding prohibiting long term anticoagulation therapy. Considering the thromboembolic risk of arterial lead placement and concern for adhesion near the ostium of the right coronary artery, we opted to proceed with transarterial lead extraction. Under general anesthesia and in the hybrid operating room, the subpectoral pocket was exposed. Thoracic aortic arch and carotid angiography was performed, and distal embolic filter wires were deployed in the right and left internal carotid arteries following anticoagulation with unfractionated heparin. Intravascular ultrasound of the left subclavian artery was performed to localize the lead entry point at the left subclavian artery and determine appropriate reference vessel sizing for stent graft placement. As the lead was withdrawn, a covered stent was deployed at the entry site as described previously. There were no neurological or vascular complications following the procedure. Reimplantation of a cardiac resynchronization device was performed electively via the left subclavian vein without any complications.

## Discussion

We report 2 cases of successful transarterial percutaneous lead removal using embolic filter protection of the cerebral circulation and stent graft placement at the arterial entry site.

Inadvertent cardiac rhythm device lead implantation via an arterial vessel is a rare event though it may be widely under recognized [1-3]. Removal of transarterially positioned leads is associated with a high thromboembolic risk as well as increased risk of bleeding from the arterial entry site. Further, in this report, lead removal posed not only risk of ventricular perforation and development of tamponade, but also potential trauma to the coronary arteries, aorta and aortic valve. Accordingly, while percutaneous lead removal is a well-established technique, it remains unclear whether it can be safely performed from the arterial system. As such, conservative management with long-term anticoagulation is usually recommended, and surgical removal has been performed when conservative management is not an option, as proposed by current guidelines [4].

Removal of chronic pacing leads percutaneously via the femoral artery [5] or via the subclavian artery has been described previously and, although there have been no reports of thromboembolic complications, the methods for removal vary and the number of reported cases is limited. Transesophageal echocardiography has been universally performed, and heparin is instituted in patients with left ventricular lead implants to minimize the risk of thrombus formation. However, given the direct communication with the systemic circulation, any lead manipulation can potentially lead to systemic embolization, preferentially in the cerebral circulation. With the technique presented herein, we utilized standard methodology employed in interventional cardiology for cerebral embolic protection and peripheral stent placement with excellent results. We demonstrate that stent deployment is readily feasible simultaneous with lead removal and provides immediate and effective hemostasis. This may provide a solution in cases where conventional or laser sheaths are utilized resulting in expansion of the arterial puncture site. A technique employing a suture-based percutaneous closure device following transarterial lead removal from the subclavian artery has also been described, yet this method may only apply to cases for which no sheath is utilized for lead removal.

To our knowledge, this is the first description of transarterial lead removal of pacing and defibrillator leads with concomitant deployment of carotid embolic protection devices and stent graft placement for hemostasis at the removal site in the subclavian artery. This approach may effectively reduce the risk of systemic thromboembolism and arterial site bleeding associated with transarterial lead removal.
